# Gut Microbiota and Mycobiota Evolution Is Linked to Memory Improvement after Bariatric Surgery in Obese Patients: A Pilot Study

**DOI:** 10.3390/nu13114061

**Published:** 2021-11-13

**Authors:** Raphaël Enaud, Sophie Cambos, Esther Viaud, Erwan Guichoux, Emilie Chancerel, Aline Marighetto, Nicole Etchamendy, Samantha Clark, Kamel Mohammedi, Daniela Cota, Laurence Delhaes, Blandine Gatta-Cherifi

**Affiliations:** 1 Centre Hospitalier de Bordeaux, CRCM Pédiatrique, CIC 1401, 33000 Bordeaux, France; raphael.enaud@chu-bordeaux.fr; 2 Centre de Recherche Cardio-Thoracique de Bordeaux, U1045, Hôpital Xavier Arnozan, University of Bordeaux, Avenue du Haut Lévêque, 33604 Pessac, France; laurence.delhaes@chu-bordeaux.fr; 3 Centre Hospitalier de Bordeaux, University of Bordeaux, FHU ACRONIM, 33000 Bordeaux, France; kamel.mohammedi@chu-bordeaux.fr; 4 Centre Hospitalier de Bordeaux, Department of Endocrinology, Diabetes and Nutrition, University Hospital of Bordeaux, 33604 Pessac, France; sophie.cambos@chu-bordeaux.fr; 5 Neurocentre Magendie, Physiopathologie de la Plasticité Neuronale, U862, INSERM, 146 rue Léo Saignat, 33076 Bordeaux, France; esther.viaud@gmail.com (E.V.); aline.marighetto@inserm.fr (A.M.); nicole.etchamendy@u-bordeaux.fr (N.E.); samantha.clark@inserm.fr (S.C.); daniela.cota@inserm.fr (D.C.); 6 BIOGECO, INRAE, University of Bordeaux, 69 route d’Arcachon, 33610 Cestas, France; erwan.guichoux@inrae.fr (E.G.); emilie.chancerel@inrae.fr (E.C.); 7 Centre Hospitalier de Bordeaux, University of Bordeaux, FHU TALISMENT, 33000 Bordeaux, France

**Keywords:** cognition, memory, metagenomics, microbiome, microbiota, mycobiota, obesity, bariatric surgery

## Abstract

Patients with obesity are known to exhibit gut microbiota dysbiosis and memory deficits. Bariatric surgery (BS) is currently the most efficient anti-obesity treatment and may improve both gut dysbiosis and cognition. However, no study has investigated association between changes of gut microbiota and cognitive function after BS. We prospectively evaluated 13 obese patients on anthropometric data, memory functions, and gut microbiota-mycobiota before and six months after BS. The Rey Auditory Verbal Learning Test (AVLT) and the symbol span (SS) of the Weschler Memory Scale were used to assess verbal and working memory, respectively. Fecal microbiota and mycobiota were longitudinally analyzed by 16S and ITS2 rRNA sequencing respectively. AVLT and SS scores were significantly improved after BS (AVLT scores: 9.7 ± 1.7 vs. 11.2 ± 1.9, *p* = 0.02, and SS scores: 9.7 ± 23.0 vs. 11.6 ± 2.9, *p* = 0.05). An increase in bacterial alpha-diversity, and *Ruminococcaceae*, *Prevotella, Agaricus*, *Rhodotorula*, *Dipodascus*, *Malassezia*, and *Mucor* were significantly associated with AVLT score improvement after BS, while an increase in *Prevotella* and a decrease in *Clostridium*, *Akkermansia,* *Dipodascus* and *Candida* were linked to SS scores improvement. We identified several changes in the microbial communities that differ according to the improvement of either the verbal or working memories, suggesting a complex gut-brain-axis that evolves after BS.

## 1. Introduction

Besides causing diabetes, cardiometabolic disease and cancer, obesity is now a recognized risk factor for cognitive disorders including memory decline [1,2,3]. However, little data is available in humans concerning the underlying mechanisms of obesity-related memory impairment and of its potential reversibility with weight loss. One potential mechanism through which obesity could affect cognitive function is modifications in gut microbiota. Indeed, gut microbiota dysbiosis is well documented in obesity [4]. Hypercaloric diets trigger robust gut microbiota disturbances (or dysbiosis) and evidence from animals and humans implies that gut microbiota affects brain structure and cognitive function [5,6]. In a recent study, a specific microbiota profile that involves aromatic amino acid and one-carbon metabolism has been significantly associated with memory and modulated by obesity [1].

Bariatric surgery (BS) is currently the most efficient anti-obesity and obesity-linked comorbidities treatment, and may even improve cognition [7,8,9,10]. BS can partially rescue the gut dysbiosis linked to obesity [4,7,10,11,12]. These gut microbiome changes contribute to the positive effects of BS, especially the metabolic improvements [12,13]. To date, few studies have investigated the impact of the longitudinal change of gut microbiota and mycobiota composition on memory after BS [10]. In this context, we aim at setting out to clarify the relationship between the evolution of the gut microbiota and mycobiota and memory performance after BS. We present here the results of a pilot study.

## 2. Materials and Methods

### 2.1. Participants

We prospectively evaluated patients with obesity aged between 18 and 65 years, followed in the Nutrition Department of the Bordeaux University Hospital, requiring a bariatric surgery planned according to the guidelines of the French Health Authority: BMI >40 kg/m^2^ or > 35 kg/m^2^ in the presence of complications susceptible to be improved by bariatric surgery. Exclusion criteria were excessive alcohol consumption (more than 20 g per day for women, more than 30 g per day for men) or drug abuse (recent or past use), a history of stroke, cerebral radiotherapy, cerebral neurological disease, chronic digestive disease, or the presence of unbalanced dysthyroidism or a current psychotropic treatment. Anthropometric and biological data were collected, as well as scores of the assessment of cognitive functions and fecal samples for microbiota and mycobiota analysis, at the pre- (the day before surgery) and 6-month post-surgery visits. This study was approved by the ethics committee of SUD-EST VI (N° 2017-A03504-49).

### 2.2. Assessment of Cognitive Functions

Memory and learning ability in the auditory-verbal domain were assessed at the pre- and post-operative visit with the Rey Auditory Verbal Learning Test (AVLT) and one subtest of the Weschler Memory Scale, fourth edition (WMS-IV), namely the symbol span (SS) to evaluate verbal and working memory respectively [14,15,16]. A postoperative score amelioration of at least 5% was considered as an improvement and allowed us to classify patients in responders for AVLT or SS, and non-responders.

### 2.3. Fecal Sample Collection and Sequencing

Fecal microbiota and mycobiota were analyzed as previously reported [17]. Briefly, fecal samples were collected at baseline and six months after surgery and stored at −80 °C until analysis. DNA extraction was performed using QIAamp^®^ PowerFaecal^®^ DNA kit (QIAgen^®^, Valencia, CA, USA). A first step of mechanical lysis (2 cycles of 30 s at 7000 rpm on Precellys evolution) was added to the chemical lysis of the kit as previously described [17]. The gut microbiota and mycobiota composition of samples were assessed, respectively, by using the V3-V4 regions of the bacterial 16S rRNA encoding gene and the internal transcribed spacer 2 (ITS2) region of the fungal rDNA. The respective primers used to amplify these loci were as follows: 16S-forward, TACGGRAGGCAGCAG; 16S-reverse, CTACCNGGGTATCTAAT; ITS2-forward, GTGARTCATCGAATCTTT; and ITS2-reverse, GATATGCTTAAGTTCAGCGGGT. Sequencing (2 × 250 bp paired-end) was performed on MiSeq sequencer (Illumina^®^, San Diego, CA, USA) at the PGTB platform (INRAe, University of Bordeaux, Cestas, France).

### 2.4. Bacterial and Fungal Sequence Analyses

The bacterial and fungal reads were demultiplexed; 16S and ITS2 primers were removed using CutAdapt, with no mismatch allowed within the primer sequences. All samples were processed through the DADA2 pipeline in R (version 4.0.3) for quality filtering and trimming, dereplication, and merging of paired-ends reads [18,19]. According to a recent evaluation [20], only forward sequences were analyzed with DADA2 and no filter other than the removal of low-quality and chimeric sequences were applied for characterizing the fungal community. Two distinct ASV tables were constructed, and taxonomy was assigned from the Silva database (release 138) for bacterial ASVs and the Unite database (release 8.2) for fungal ASVs. We used mock communities to avoid a non-efficient sequencing experiment, and negative controls to identify and remove potential reagent contaminants of bacterial and fungal microbiota with the microDecon R package [21]. The final median read counts were 69861 (interquartile rang [IQR]: 49374; 106078) for 1403 bacterial ASVs and 7666 (2566; 14238) for 742 fungal ASVs. The 16S rRNA gene and ITS2 sequences have been submitted to the European Nucleotide Archive (Accession N° PRJEB42057).

### 2.5. Statistical Analyses

Results were expressed as mean (± standard deviation (SD)) for parametric variables, median and [IQR] for non-parametric variables or in absolute values and percentage (n/N (%)) for categorical variables. A non-parametric Wilcoxon–Mann–Whitney test was used to compare quantitative variables between groups. Correlations were calculated using the Spearman method. The McNemar’s test and Wilcoxon signed-rank test were used as statistical tests for paired nominal data and for quantitative data, respectively. Statistical analysis was performed with the R studio program (version 1.3.1056 for Windows^TM^); correction for multiple-testing was performed using the Benjamini-Hochberg false discovery rate (FDR) procedure, a *p*-value or FDR adjusted *p*-value equal to or less than 0.05 was considered statistically significant.

For microbiota and mycobiota analysis, alpha-diversity metrics (ACE, Simpson and Shannon indexes) were generated by using the phyloseq R package. For cross-sectional analyses, at a specific time, significant differences in alpha-diversity were determined using the Wilcoxon rank sum. Microbiota and mycobiota compositions were longitudinally compared using the DESeq2 and GAMLSS-BEZI models, accordingly to time and/or memory score evolution [22,23,24]. Between sample beta-diversity differences (measured using Bray Curtis dissimilarity) were tested using a permutational multivariate ANOVA (PERMANOVA) from vegan R package with 10,000 permutations, while accounting for individual identity as a covariate.

## 3. Results

We prospectively evaluated 13 obese patients (11 females, mean age: 48 ± 12 years) before and 6 months after BS (sleeve gastrectomy: *n* = 7, Roux-en-Y-Gastric-Bypass (RYGBP): *n* = 6). Anthropometric and biological data of patients at baseline are summarized in Table 1. Gut microbiota composition at baseline was dominated by Firmicutes (50%), followed by Bacteroidetes (39%) and Actinobacteria (5%). Proteobacteria represented 4% of the bacterial ASVs. Bacterial genera were dominated by *Bacteroides* (21%), followed by *Megasphaera* (6%), *Alistipes* (5%), *Blautia* (4%), *Prevotella* (4%) and *Streptococcus* (4%). *Faecalibacterium* represented 2% of the bacterial ASVs. Fungal genera were dominated by *Saccharomyces* (34%), followed by *Penicillium* (20%), *Debaryomyces* (14%), *Agaricus* (12%), *Dipodascus* (2%) and *Malassezia* (2%).

Six months after BS, body weight and body mass index were significantly decreased, as compared to pre-surgery (115.6 ± 4.3 vs. 92.4 ± 2.7 kg, *p* < 0.05, and 43.1 ± 1.3 vs. 34.2 ± 0.8, kg/m², *p* < 0.05, respectively). AVLT and SS scores were significantly improved after BS (AVLT scores: 9.7 ± 1.7 vs. 11.2 ± 1.9, *p* = 0.02, and SS scores: 9.7 ± 23.0 vs. 11.6 ± 2.9, *p* = 0.05). A postoperative score amelioration of at least 5% was considered as an improvement and allowed us to classify patients in responders for AVLT (*n* = 9) or SS (*n* = 8) and non-responders (*n* = 4 and 5 for AVLT and SS, respectively). Clinical and biological characteristics of patients who improved or not as reflected in the tests are summarized in Table 1. AVLT responders exhibited less history of type 2 diabetes (*p* = 0.05), while a trend towards a lower HOMA index was observed for SS responders (*p* = 0.09).

**Figure 1:**
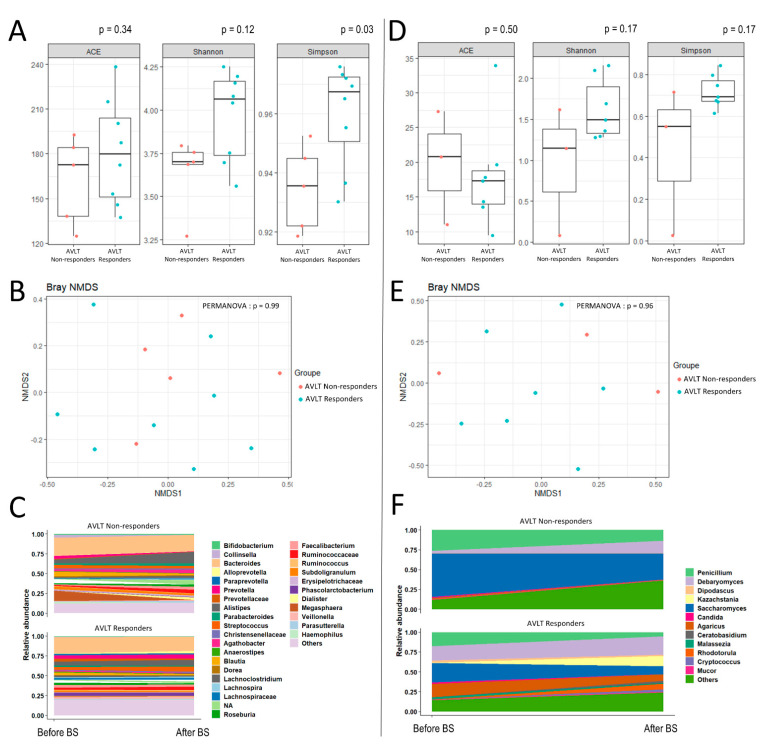
Alpha-diversity indexes, beta-diversity and longitudinal modulation of microbiota and mycobiota according to the evolution of verbal memory, at six months after bariatric surgery. Fecal samples were collected the day before surgery and six months after bariatric surgery (BS). (**A**,**B**) show respectively the alpha-diversity indexes and beta-diversity in post-BS, according to an improvement or not of AVLT score at six months post-BS. Beta diversity, which assesses differences in microbiota composition between samples according to lung function at baseline, using a Non-metric Multidimensional Scaling (NMDS) ordination method with Bray–Curtis distance metric. (**C**) shows the longitudinal evolution of bacterial microbiota using GAMLSS-BEZI models, according to an improvement or not of AVLT score at six months post-BS. (**D**–**F**) illustrate the same parameters for the mycobiota.

In AVLT responders, bacterial alpha-diversity (Simpson index) was significantly higher after BS when compared to non-responders (Figure 1A). Beta-diversity was not significantly different between AVLT responder and nonresponder groups. Table 2 summarizes significant changes over time in bacterial and fungal compositions after bariatric surgery in ALVT responder patients, compared to AVLT non-responders. A significant increase in *Ruminococcus* and *Prevotella* sp. was associated with an improvement of AVLT scores (Figure 1C, Table 2). Regarding the mycobiota, *Agaricus*, *Rhodotorula*, *Dipodascus*, *Malassezia*, and *Mucor* were significantly associated with AVLT score improvement after BS (Figure 1F, Table 2).

**Figure 2:**
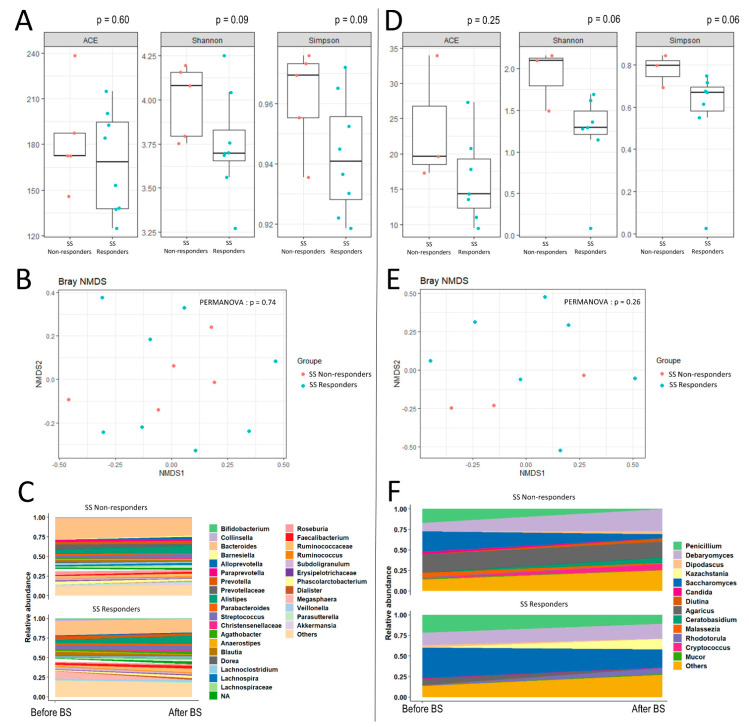
Alpha-diversity indexes, beta-diversity and longitudinal modulation of microbiota and mycobiota according to the evolution of working memory, at six months after bariatric surgery. (**A**,**B**) show respectively the alpha-diversity indexes and beta-diversity in post-BS, according to an improvement or not of SS score at six months post-BS. Beta diversity which assesses differences in microbiota composition between samples according to lung function at baseline, using a Non-metric Multidimensional Scaling (NMDS) ordination method with Bray–Curtis distance metric. (**C**) shows the longitudinal evolution of bacterial microbiota, according to an improvement or not of SS score at six months post-BS. (**D**–**F**) illustrate the same parameters for the mycobiota.

In SS responders, we also found clear changes in the bacterial and fungal communities (Figure 2, Table 3). There was a trend in reduced fungal richness and diversity (Shannon and Simpson indexes, Figure 2C) in patients with improved SS scores after BS compared to those who did not. Beta-diversity was not significantly different between SS responder and nonresponder groups. An increase in *Prevotella* and *Parabacteroides* was significantly associated with SS improvement, while a decrease in *Clostridium* and *Akkermansia* species was linked to working memory improvement. We also identified a significant decrease in *Saccharomyces* (*Dipodascus* and *Candida* genera) associated with SS score and working memory improvements (Figure 2F, Table 3).

## 4. Discussion

We longitudinally assessed cognitive functions and characterized the fungal and bacterial compositions of fecal samples from morbidly obese patients before and six months after BS in order to evaluate associations between gut microbiota-mycobiota evolution and memory domain improvements after BS.

In this approach, several points had to be taken into account. First, obesity itself was associated with different disturbances of the microbiota, such as a decrease in alpha-diversity, a decrease in Bacteroidetes at the phyla level, or in *Bacteroides faecichinchillae*, *Bacteroides thetaiotaomicron*, *Blautia wexlerae*, *Clostridium bolteae* and *Flavonifractor plautii*, *Akkermansia muciniphila* at the species level, and an increase in *Enterobacter* [25,26]. In addition, modifications in the composition of the microbiota have been observed after bariatric surgery due to various factors such as changes in food choices, level of hormones or pH of the stomach [4,7,10,11,12]. For example, we identified a decrease in *Blautia* spp. and *Dorea* spp. as previously described in post-BS patients [4,11,27]. Lastly, intense weight loss in obese patients, independent of BS, has been shown to be associated with improved cognitive function, along with increased gray matter volume in the inferior frontal gyrus and hippocampus [27]. Thus, by longitudinally following patients before and after BS and comparing patients who improved their memory function after BS with those who did not, we were able to limit these confounding factors in order to more closely examine the association between the evolution of microbiota and memory function after BS.

We identified several changes in the microbial communities that differ according to the improvement of either AVLT or SS tests. The improvement in AVLT scores was significantly associated with higher bacterial alpha-diversity (Simpson index) after BS compared to non-responders. An elevated alpha-diversity of the gut microbiota is often found to be an indicator of good health, particularly with regard to cognitive function [28,29]. Conversely, alpha-diversity was found to be reduced in pathologies associated with cognitive disturbances, such as obesity, but also in type 2 diabetes and patients with insulin resistance, while these later conditions are related to hippocampal memory disorders [30,31]. Interestingly, less history of type 2 diabetes was observed in AVLT responder patients (Table 1), which may suggest the role of insulin resistance in memory disorders persisting after BS.

Regarding the composition of the microbiota, a significant increase in *Ruminococcus* and *Prevotella* sp. was associated with improved AVLT scores, in agreement with the positive association between these taxa and verbal memory in both obese and normal weight subjects [1]. An increase in *Prevotella* and *Parabacteroides* was also significantly associated with SS improvement, while a decrease in the *Clostridium* and *Akkermansia* species was linked to working memory improvement. Of note, a positive association of *Clostridium* with working memory has been reported in obese patients, as well as a positive association of both *Clostridium* and *Akkermansia* with higher memory scores in mice after microbiota transplantation [1]. *Parabacteroides* may also be involved in the gut-brain axis and its abundance was positively correlated with food behavior in a recent mouse model [6]. We also identified a significant decrease in *Candida* genera associated with SS score and working memory improvements. Interestingly, we can mention that most of these bacterial and fungal taxa referred to above have been studied in chronic inflammatory bowel disease (IBD). Notably, intestinal inflammation in IBD is instead associated with an increase in *Candida* and a decrease in *Parabacteroides* and *Ruminococcus* [32,33]. A common feature of metabolic diseases is the presence of chronic low-grade inflammation and recent reports suggest that neuroinflammation is an important causal mechanism in cognitive decline. One hypothesis is that the digestive microbiota contributes to this inflammation, both locally and systemically, through several pathways including direct inflammatory stimulation, the production of neurotransmitters and pro-inflammatory metabolites, and the loss of immune-regulatory function [34]. The study of the association between the evolution of microbiota, local and systemic inflammation and memory functions after BS therefore deserves further investigation.

We identified several changes in the bacterial and fungal kingdoms that differ according to the improvement of either the verbal memory or the working memory, such as the *Christensenellaceae*, whose increased amounts are associated with an improvement of SS scores (Figure 1D and Figure 2D, Table 3), while the gut relative abundance of *Christensenellaceae* has been related to host health and negatively related to body mass index [35]. However, *Christensenellaceae* was decreased in AVLT responders, and could illustrate divergent associations between microbiota and memory domain that may exist [1] and could be pointed out with longitudinal studies in post-BS.

As previously reported, the type of surgery can influence both microbiota changes [7,36] and short-term improvements in cognitive function [37]. In addition, the clinical evolution of the obese patients as well as that of their gut microbiota-mycobiota is probably not stabilized at six months post-operation. Obviously, obesity comorbidities, such as type 2 diabetes, may also interfere with the evolution of gut microbiota-mycobiota and/or cognitive functions [38], but other parameters like the duration of obesity are also involved.

Some limitations of our study should be noted. We applied targeted metagenomics based on rDNA signatures that is not designed to assess metabolic functions. We characterized only 13 patients before and six months post-BS in this pilot study. Therefore, studies on larger cohorts that differ in age, gender, duration of obesity and insulin sensitivity with longer follow-up are warranted to confirm our results and to more deeply investigate these links.

Despite these limitations, our longitudinal study highlights the crucial role of gut microbiota-mycobiota in somatic and cognitive human health, suggesting a complex gut-brain-axis ecology that evolves dynamically to adapt body and brain physiology in response to BS. These different complex mechanisms deserve to be explored further through complementary study, such as the involvement of insulin resistance or neuroinflammation in the associations found between microbiota and cognitive functions.

## Figures and Tables

**Table 1:** Characterization at baseline of patients who improved (Yes) and who did not improve (No) in AVLT and Symbol span tests.

	Overall	AVLT Responders			Symbol Span Responders		
		No	Yes	*p*	No	Yes	*p*
N	13	9 (69%)	4 (31%)		8 (62%)	5 (38%)	
Female	11 (85%)	9 (100%)	2(50%)	0.53	7 (75%)	5 (100%)	0.99
Age (years)	48 ± 12	47 ± 13	44 ± 12	0.70	45 ± 15	45 ± 10	0.92
Obesity duration (years)	25 ± 8	29.3 ± 6.0	22.7 ± 8.2	0.20	30 ± 6	22 ± 7	0.10
BMI (kg/m^2^)	43 ± 5	43 ± 6	43 ± 4	0.96	44 ± 5	43 ± 5	0.75
History of diabetes	4 (31%)	3 (33%)	1 (25%)	0.05	2 (25%)	2 (40%)	0.99
HOMA	4 [2; 7]	12 [3; 66]	4 [2; 5]	0.14	5 [4; 66]	3 [2; 5]	0.09
CRP (mg/L)	6 [4; 13]	4 [3; 11]	9 [5; 15]	0.22	9 [5; 13]	5 [3; 15]	0.33
Weight loss (%)	18 [14; 25]	21 [11; 34]	18 [14; 22]	0.76	18 [13; 28]	17 [14; 22]	0.88
Vit B1 postop	149 [128; 178]	156 [121; 191]	149 [133; 175]	0.99	156 [127; 173]	149 [128; 186]	0.62
Vit B12 postop	361 [314; 520]	381 [320; 442]	361 [306; 663]	0.99	343 [295; 390]	379 [320; 754]	0.57
Type of surgery							
SG		5 (56%)	2 (50%)	0.99	5 (62%)	2 (40%)	0.59
Gastric bypass		4 (44%)	2 (50%)		3 (37%)	3 (60%)	

Results were expressed as mean (± SD), median ([IQR]) or values (%). AVLT: Rey Auditory Verbal Learning Test; BMI: body Mass Index; HOMA: Homeostasic model assessment; SG: sleeve gastrectomy; CRP: C reactive protein.

**Table 2:** Significant bacterial and fungal changes after bariatric surgery in AVLT responder patients, compared to AVLT Non-responders.

Kingdom	Phylum	Class	Order	Family	Genus	Odds Ratio (Log)	95% CI	FDR Adjusted *p*-Value
Bacteria	Firmicutes	Negativicutes	Selenomonadales	Veillonellaceae	Dialister	−1.7	(−1.9, −1.5)	<0.0001
Bacteria	Firmicutes	Negativicutes	Selenomonadales	Veillonellaceae	Megasphaera	−1.6	(−1.6, −1.6)	<0.0001
Bacteria	Actinobacteria	Actinobacteria	Bifidobacteriales	Bifidobacteriaceae	Bifidobacterium	−1	(−1.7, −0.3)	0.03
Bacteria	Firmicutes	Clostridia	Clostridiales	Peptostreptococcaceae	Romboutsia	−0.6	(−0.6, −0.6)	<0.0001
Bacteria	Firmicutes	Clostridia	Clostridiales	Christensenellaceae	Christensenellaceae	−0.6	(−0.8, −0.3)	0.005
Bacteria	Firmicutes	Clostridia	Clostridiales	Lachnospiraceae	NA	−0.6	(−0.9, −0.3)	0.01
Fungi	Mucoromycota	Mucoromycetes	Mucorales	Mucoraceae	Mucor	0.2	(0.2, 0.2)	<0.0001
Bacteria	Firmicutes	Clostridia	Clostridiales	Lachnospiraceae	Anaerostipes	0.3	(0.2, 0.4)	0.005
Fungi	Ascomycota	Saccharomycetes	Saccharomycetales	Saccharomycetal	Candida	0.3	(0.3, 0.3)	<0.0001
Bacteria	Firmicutes	Clostridia	Clostridiales	Lachnospiraceae	Lachnospiraceae	0.7	(0.3, 1.1)	0.01
Bacteria	Bacteroidetes	Bacteroidia	Bacteroidales	Barnesiellaceae	Barnesiella	0.7	(0.5, 1)	<0.0001
Bacteria	Firmicutes	Clostridia	Clostridiales	Lachnospiraceae	Lachnospira	0.8	(0.3, 1.4)	0.04
Bacteria	Firmicutes	Clostridia	Clostridiales	Ruminococcaceae	NA	0.9	(0.4, 1.3)	0.006
Bacteria	Bacteroidetes	Bacteroidia	Bacteroidales	Prevotellaceae	Alloprevotella	1	(1, 1)	<0.0001
Fungi	Basidiomycota	Microbotryomycetes	Sporidiobolales	Sporidiobolaceae	Rhodotorula	1	(1, 1)	<0.0001
Bacteria	Bacteroidetes	Bacteroidia	Bacteroidales	Prevotellaceae	Prevotella	1.2	(1, 1.4)	<0.0001
Fungi	Basidiomycota	Malasseziomycetes	Malasseziales	Malasseziaceae	Malassezia	1.5	(1.4, 1.6)	<0.0001
Fungi	Ascomycota	Saccharomycetes	Saccharomycetales	Dipodascaceae	Dipodascus	2.6	(2.6, 2.6)	<0.0001
Fungi	Basidiomycota	Agaricomycetes	Agaricales	Agaricaceae	Agaricus	4.2	(4.2, 4.2)	<0.0001

Taxa are ranked in increasing order of odd ratio. CI: Confidence Interval; NA: Not Assigned.

**Table 3:** Significant bacterial and fungal changes after bariatric surgery in SS responder patients, compared to SS Non-responders.

Kingdom	Phylum	Class	Order	Family	Genus	Odds Ratio (Log)	95% CI	FDR Adjusted *p*-Value
Fungi	Basidiomycota	Malasseziomycetes	Malasseziales	Malasseziaceae	Malassezia	−3.8	(−3.8, −3.7)	<0.0001
Bacteria	Verrucomicrobia	Verrucomicrobiae	Verrucomicrobiales	Akkermansiaceae	Akkermansia	−2.6	(−2.6, −2.6)	<0.0001
Fungi	Ascomycota	Saccharomycetes	Saccharomycetales	Dipodascaceae	Dipodascus	−2.5	(−2.5, −2.5)	<0.0001
Bacteria	Firmicutes	Negativicutes	Selenomonadales	Veillonellaceae	Megasphaera	−2.4	(−2.4, −2.3)	<0.0001
Fungi	Ascomycota	Saccharomycetes	Saccharomycetales	Saccharomycetal	Candida	−1.7	(−1.7, −1.7)	<0.0001
Bacteria	Bacteroidetes	Bacteroidia	Bacteroidales	Prevotellaceae	Prevotellaceae	−1.6	(−2.6, −0.7)	0.0084
Bacteria	Bacteroidetes	Bacteroidia	Bacteroidales	Barnesiellaceae	Barnesiella	−1.4	(−1.6, −1.2)	<0.0001
Bacteria	Bacteroidetes	Bacteroidia	Bacteroidales	Prevotellaceae	Alloprevotella	−1.2	(−1.2, −1.2)	<0.0001
Fungi	Basidiomycota	Agaricomycetes	Agaricales	Agaricaceae	Agaricus	−1.1	(−1.1, −1.1)	<0.0001
Bacteria	Bacteroidetes	Bacteroidia	Bacteroidales	Prevotellaceae	Paraprevotella	−1	(−1.6, −0.3)	0.0315
Bacteria	Firmicutes	Clostridia	Clostridiales	Lachnospiraceae	Lachnospira	−1	(−1.4, −0.5)	0.0062
Bacteria	Firmicutes	Clostridia	Clostridiales	Clostridiacea	Clostridium	−0.8	(−1.1, −0.5)	0.0016
Fungi	Mucoromycota	Mucoromycetes	Mucorales	Mucoraceae	Mucor	−0.5	(−0.5, −0.4)	<0.0001
Bacteria	Firmicutes	Clostridia	Clostridiales	Lachnospiraceae	Anaerostipes	0.2	(0.1, 0.4)	0.0148
Bacteria	Firmicutes	Clostridia	Clostridiales	Christensenellaceae	Christensenellaceae	0.4	(0.1, 0.7)	0.0315
Bacteria	Bacteroidetes	Bacteroidia	Bacteroidales	Tannerellaceae	Parabacteroides	0.7	(0.2, 1.2)	0.0315
Bacteria	Firmicutes	Negativicutes	Selenomonadales	Veillonellaceae	Dialister	0.8	(0.6, 1)	<0.0001
Bacteria	Bacteroidetes	Bacteroidia	Bacteroidales	Prevotellaceae	Prevotella	1.4	(1.3, 1.6)	<0.0001
Bacteria	Bacteroidetes	Bacteroidia	Bacteroidales	NA	NA	1.6	(1, 2.2)	0.0002

Taxa are ranked in increasing order of odd ratio. CI: Confidence Interval; NA: Not assigned.

## Data Availability

The 16S rRNA gene and ITS2 sequences have been submitted to the European Nucleotide Archive (Accession N° PRJEB42057). Other datasets generated during and/or analyzed during the current study are not publicly available but are available from the corresponding author on reasonable request.
